# Denser Markers and Advanced Statistical Method Identified More Genetic Loci Associated with Husk Traits in Maize

**DOI:** 10.1038/s41598-020-65164-0

**Published:** 2020-05-18

**Authors:** Zhenhai Cui, Haixiao Dong, Ao Zhang, Yanye Ruan, Siqi Jiang, Yan He, Zhiwu Zhang

**Affiliations:** 1 College of Biological Science and Technology, Liaoning Province Research Center of Plant Genetic Engineering Technology, Shenyang Key Laboratory of Maize Genomic Selection Breeding, Shenyang Agricultural University, Shenyang, 110866 China; 2 Dept. of Crop and Soil Sciences, Washington State University, Pullman, WA 99164 USA; 3 College of Plant Sciences, Jilin University, Changchun, 130062 China; 4 National Maize Improvement Center of China, Beijing Key Laboratory of Crop Genetic Improvement, China Agricultural University, Beijing, 100094 China

**Keywords:** Genome-wide association studies, Genome-wide association studies, Plant breeding

## Abstract

The husk—the leaf-like outer covering of maize ear—has multiple functions, including protecting the ear from diseases infection and dehydration. In previous studies, we genotyped an association panel of 508 inbred lines genotyped with a total of ~550,000 SNPs (Illumina 50 K SNP Chip and RNA-seq). Genome-Wide Association Studies (GWAS) were conducted on four husk traits: husk length (HL), husk layer number (HN), husk thickness (HT), and husk width (HW). Minimal associations were identified and none of them passed the *P-*value threshold after a Bonferroni multiple-test correction using a single locus test in framework of mixed linear model. In this study, we doubled the number of SNPs (~1,250,000 in total) by adding GBS and 600 K SNP Chip. GWAS, performed with the recently developed multiple loci model (BLINK), revealed six genetic loci associated with HN and HT above the Bonferroni multiple-test threshold. Five candidate genes were identified based on the linkage disequilibrium with these loci, including *GRMZM2G381691* and *GRMZM2G012416*. These two genes were up-regulation and down-regulation in all husk related tissues, respectively. *GRMZM2G381691* associated with HT encoded a CCT domain protein, which expressed higher in tropical than temperate maize. *GRMZM2G012416* associated with HN encoded an Armadillo (ARM) repeat protein, which regulated GA signal pathway. These associated SNPs and candidate genes paved a path to understand the genetic architecture of husk in maize.

## Introduction

Husk is the outer membranous of fruits or seeds, carrying multiple functions such as protecting the ear from diseases infection and dehydration. Maize husk exists long before domestication ancestor teosinte in Mexico sometime between ten to five thousand years ago. Teosinte only has a handful kernels, while modern maize could have hundreds of kernels. Teosinte kernels are encased in a hard shell, which evolved to maize cob. The common feature is that kernels of both teosinte and maize are wrapped with husk. Although husk also produce carbohydrates through the process of photosynthesis^1^ similar to leaves, the primary function is to provide living condition for kernels to grow, such as maintaining appropriate moisture. The secondary function is to protect kernels from attacks, such as from birds and pests damage, and pathogen infection^2–7^.

Compared with the foliar leaves that initiate from the shoot apical meristem (SAM), husks generate from the lateral meristem^8^. The maize foliar leaves present a complete C_4_ photosynthetic pathway. In contrast, although husks operate biologically as a C_4_-like photosynthetic pathway, their CO_2_ assimilation rate is inefficient and they exhibit a non-Kranz anatomy^1^. Husk surface area is closely related to the amount of cell-wall components, such as hemicellulose and cellulose^9^. Husks consist of multiple layers, typically ranging from 6 to 19 in inbreds and single-cross hybrids^10^. Husk layer number was found to be highly related to tassel branch number^11^.

Husks are commonly used as by-products in addition to kernels, including animal feed^12^, fiber for making papers^13,14^, and anthocyanins for food and cloth pigment^15^. With machine harvesting, the most economic impact of husk is the harvestability. In addition to the primary and the secondary function, husk must be dried fast enough so that grain kernels can be harvested at low cost. Thus, appropriate maize husk architecture is critically important. Measuring and breeding of this complex trait is challenge. Grain moisture content at harvesting time is determined by several husk traits, such as the husk thickness^16^, layer number^17^, tightness^18^, and husk moisture content^19^. Understanding the genetic architecture of these component traits is beneficial to understand harvestability.

The earliest effort of mapping quantitative trait loci (QTLs) underlying husk traits can be traced back to early 2000s in research on resistance to ear feeding insect and invasion^4^. Husk coverage and tightness were identified to be related to ear aflatoxin contamination. F2–3 populations were used to map QTLs. Multiple markers were identified to be significantly associated with husk tightness. These markers located on chromosomes 1 S, 1 L, 3 L, and 7 L. The marker on 3 L accounted for 12.7% of the variation and the rest were less than 10%. In 2010s, experiments have been conducted to map genes underlying husk traits. In 2018, linkage analysis was conducted for three husk traits^20^ using three maize recombinant inbred line (RIL) populations. The three traits are Husk Length (HL), Husk layer Number (HN), and Husk Width (HW), The study found 21 quantitative trait loci (QTL). Husk morphology varies widely among different maize inbred lines^10,20–22^. In 2016, the first GWAS was conducted with 253 inbred lines and 3 K markers^22^ and identified 24 markers associated with HN using the threshold of *P* < 0.001 without multiple test correction. At end of the same year the second GWAS was conducted with both number of lines and markers increased (508 lines with 0.5 M markers)^21^. The study identified 9 markers associated with HN, HW, and Husk Thickness (HT) at *P*  <  1.04 × 10^−5^ without multiple test correction. Both the GWAS studies did not find any significant markers using a threshold of α = 0.01 after Bonferroni multiple test correction^23^.

The objective of this study was to further increase number of markers and use recently developed GWAS method (BLINK) to identify associated markers for husk traits at a stringent threshold, such as α = 0.01 after Bonferroni multiple test correction. The number of markers was increased from 0.5 M to 1.25M^25^. Compared with the previous GWAS method (mixed linear model using a single loci test, the newly developed statistical method, BLINK (Bayesian-information and Linkage-disequilibrium Iteratively Nested Keyway), has higher statistical power than MLM because it replaces the single loci test with a multiple loci test^24^. As a result, we identified a new series of candidate genes associated with husk traits. This new information provides a useful resource for further functional studies aimed at understanding the molecular pathways involved in husk growth and development.

## Results

The dense maker of 1.25 M SNPs were compared with the 0.5 M SNPs used in previous study. The common SNPs (0.47 M) were named as sparse markers and used to evaluate the impact of marker density. Both the dense markers and the sparse makers were analyzed with MLM and BLINK to evaluate the impact of methods. The results from the optimum combination (dense marker and BLINK) were used for further analyses, including candidate genes.

### Genetic loci associated with husk traits

Based on RNA-seq and 50 K SNP Chip, Yang(2014)^26^ combined an integrated map for this association panel. Recently, adding GBS and 600 K SNP array data, the genotype enlarged to 1.25M^25^. To intersect previous marker from dense markers, we match the previous markers with dense markers to obtain 0.47 M SNPs. The association tests on dense and sparse markers were performed on four husk morphogenesis related traits: HL, HN, HT, and HW using BLINK.

**Figure 1:**
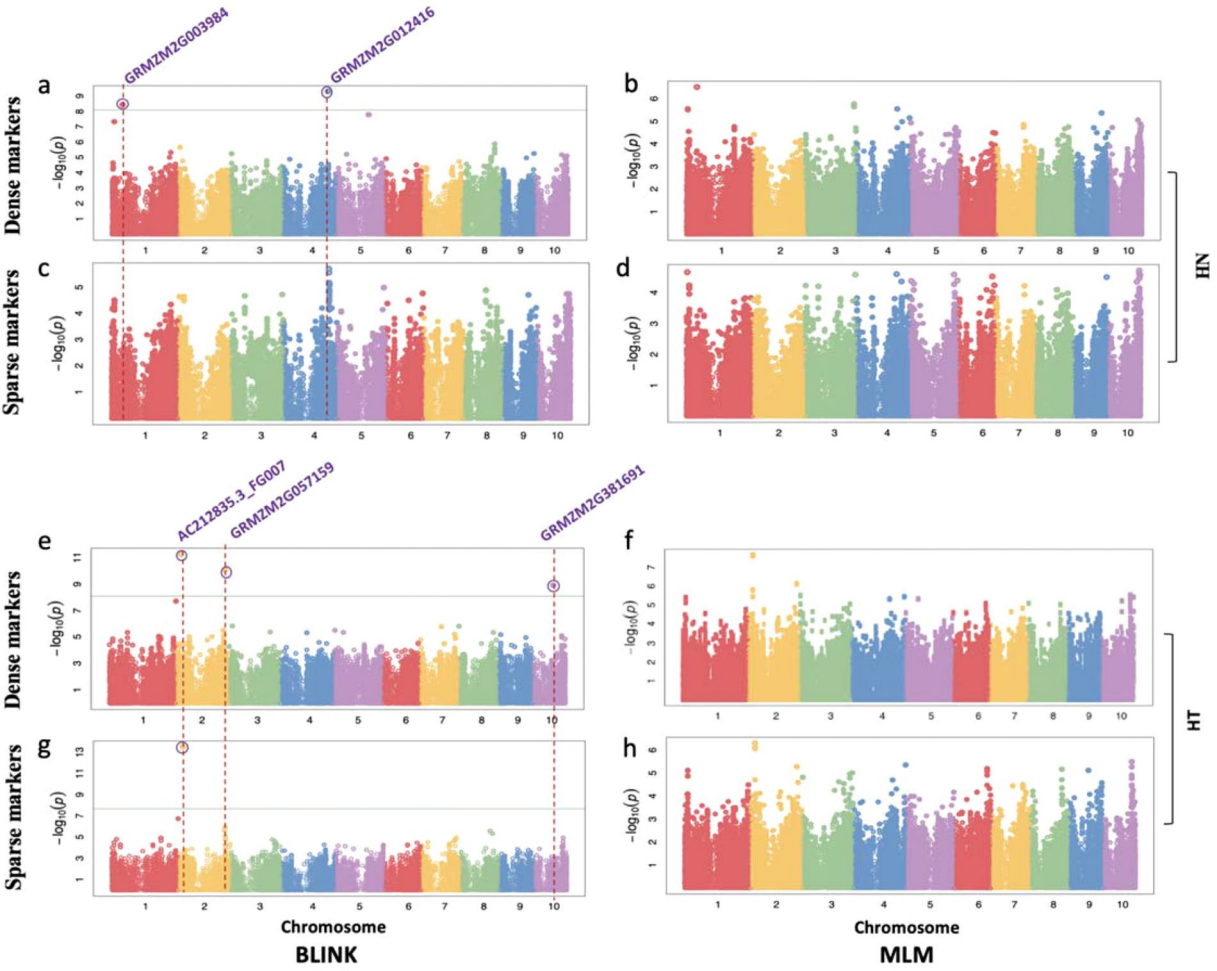
Manhattan plots with dense and sparse markers by BLINK and MLM of husk layer number and husk thickness in maize. The Manhattan plots on the left panel display the *P-*values of the SNPs, categorized by chromosome and position along the chromosome, associated with Husk layer Number (HN) and Husk Thickness (HT). The *P*-values were calculated using BLINK and MLM software. The physical positions of all significant SNPs are displayed as the vertical dashed lines and the candidate genes for the husk traits are listed at the top of each line. The horizontal green line represents the Bonferroni multiple test threshold corresponding to a type I error of 1% with dense markers and sparse markers (*p* < 7.98 × 10^−9^ and *p* < 2.11 × 10^−8^). Six SNPs (purple circles) were identified as significantly associated with husk thickness and were located in or near the five candidate genes. (**a)** Mahattan plot of HN with dense markers by BLINK. (**b**) Mahattan plot of HN with dense markers by MLM. (**c**) Mahattan plot of HN with sparse markers by BLINK. (**d**) Mahattan plot of HN with sparse markers by MLM. (**e**) Mahattan plot of HT with dense markers by BLINK. (f) Mahattan plot of HT with dense markers by MLM. (g) Mahattan plot of HT with sparse markers by BLINK. (h) Mahattan plot of HT with sparse markers by MLM.

No SNP passed the default threshold after Bonferroni multiple test correction for HL and HW (Fig. S1). The GWAS results of HN and HT on dense and sparse marker with BLINK are displayed in Fig. 1 and Table 1. Although no significant SNP was found by MLM method, we demonstrated the Manhattan plot of HN and HT by MLM as comparison. In total, six significant SNPs were detected for HN and HT by BLINK. In dense markers, we detected two and three significant SNPs for HN and HT. Percentage of phenotypic variation explained by the identified SNPs for HN and HT were 20.85 and 57.33%. Only one significant SNP(SNP3) was derived from both newer and older sequencing platforms (GBS and RNA-seq). The other four significant SNPs were derived from new sequencing platforms (GBS or 600 K) that were unavailable at the time of our previous study^22^. In sparse markers, we only detected one significant SNP for HT, which was overlapped with dense markers for HT by BLINK.

**Table 1:** Attributes of the five SNPs associated with husk layer number and husk thickness.

SNP	Trait^a^	Chr	Position (bp)	Allele^b^	PVE^c^	MAF^d^	*P*-value	Sequencing platform^e^
SNP1	HN	1	48923085	C/G	6.14	0.06	3.63E-9	600 K
SNP2	HN	4	204094290	A/G	7.34	0.26	4.93E-10	600 K
Total^f^					20.85			
SNP3	HT	2	21007733	T/C	8.79	0.11	6.15E-11	GBS, RNA-seq
SNP4	HT	2	221361730	T/G	10.39	0.06	1.09E-11	GBS
SNP5	HT	10	94253633	C/T	13.17	0.18	1.19E-09	600 K
Total^f^					57.33			

^a^Husk layer Number (HN) and Husk Thickness (HT).

^b^Major/minor allele, underlined bases are the favorable alleles.

^c^Percentage of phenotypic variation explained by the additive effect of the single significant SNP (PVE).

^d^Minor Allele Frequency (MAF).

^e^GBS = genotyping-by-sequencing; 600 K = SNP array with ~600 K markers; RNA-seq = RNA sequencing.

^f^Total percentage of phenotypic variation explained by all significant SNPs.

### Genotype effects of significant SNPs associated with husk traits

**Figure 2:**
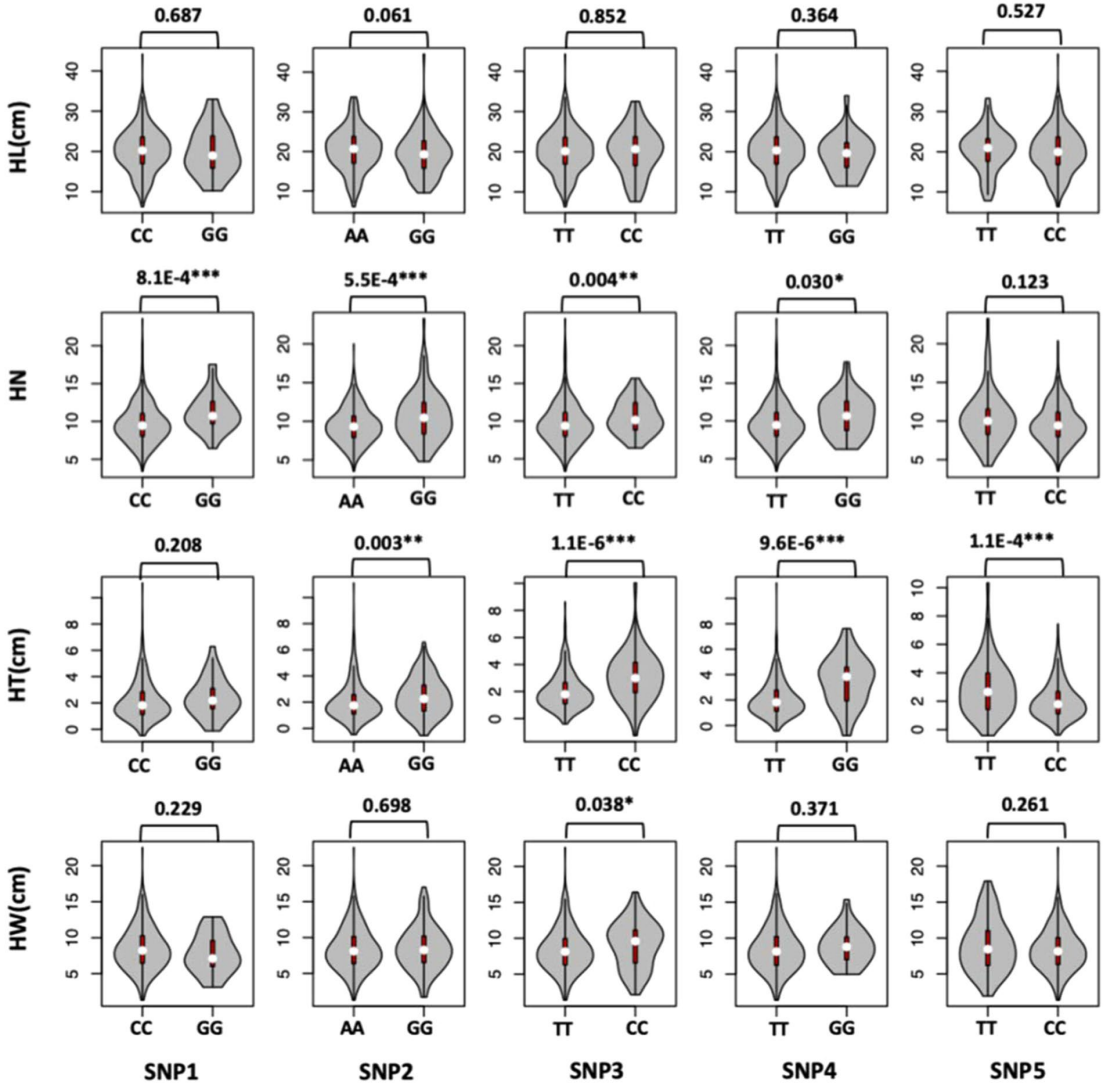
Violin plots of allelic effects of five SNPs associated with husk traits. In a violin plot, the inner red box represents the interquartile range. The central white dot represents the median value. The outer gray shape on each side represents all measured data points and the thickness represents the probability density of the data. The *P*-values of the two allelic effects of the four husk traits [Husk Length (HL), Husk layer Number (HN), Husk Thickness (HT), Husk Width (HW)] are exhibited above each small plot. *Significant at *P* ≤  0.05; **Significant at *P*  ≤ 0.01; ***Significant at *P* ≤  0.001.

The phenotypic distribution of genotypes of the twelve associated SNPs are displayed for HN and HT. The differences between the two homozygous genotypes were examined by linear model (LM) with principal component analysis (PCA) (Fig. 2). Although the LM results could be different from GWAS results, there is substantial agreement. For example, the SNP on Chromosome 4 (SNP2) was the most significant SNP for HN from GWAS with *P*-value of 4.93E-10. The LM on the difference between the two genotypes of SNP2 was also the most significant for HN with *P*-value of 5.5E-4. The genotype of AA was 9.64% less than GG for HN. However, with incorporating other factors, GWAS considered the SNP on chromosome 2 (SNP3) as the most significant for HW. Genotype CC was 12.21% wider than genotype TT.

## Candidate genes selection based on LD of significant SNPs

**Figure 3:**
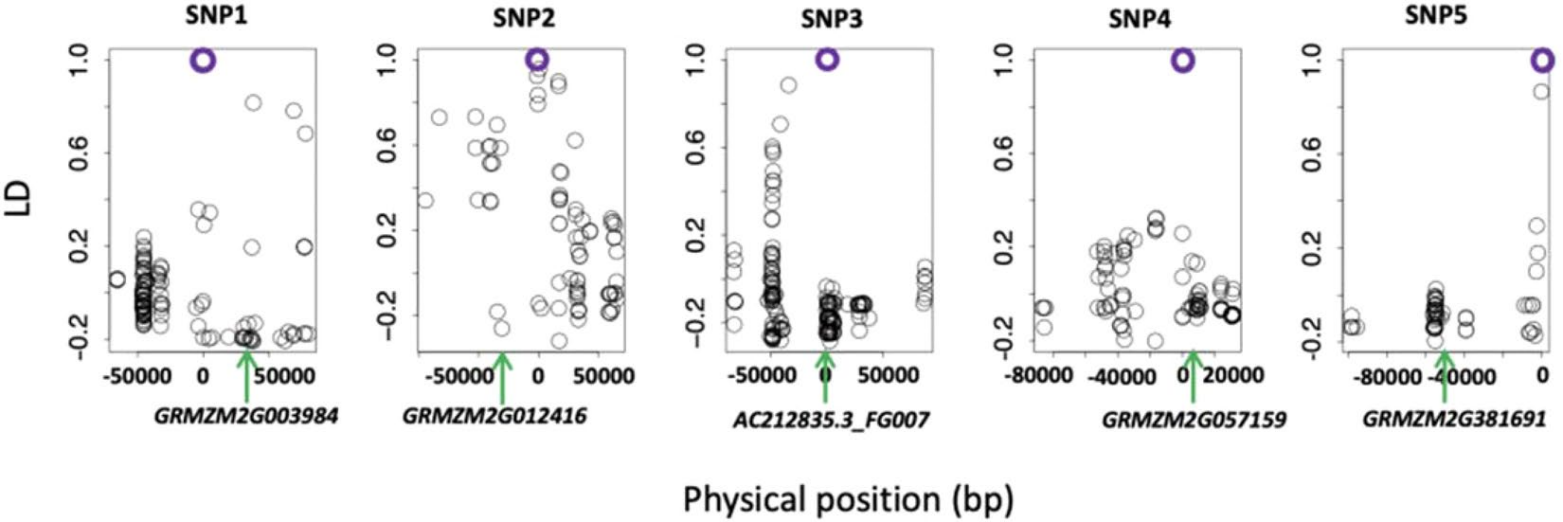
Linkage disequilibrium (**LD) decay within one million bp surrounding the five candidate QTNs**. The physical position of five significant SNPs (purple circles) associated with husk layer number and husk thickness were defined as zero in x axis. The physical position of five candidate genes were displayed with green arrows.

According to the B73 RefGen v2 (*AGPv2*), one significant SNPs for HT were located on the candidate genes, *AC212835.3_FG007*. For the other four significant SNPs, we perform the linkage disequilibrium (LD) decay within 1 Mb (Fig. 3). For SNP1, the nearest gene is *GRMZM2G003984*, which located downstream 30602 bp to SNP1(Table 2). In this location, the LD decay was less than 0.2. For SNP2, the nearest gene is *GRMZM2G012416*, which located upstream 29974 bp to SNP2. In this location, the LD decay was less than 0.3. For SNP4 and SNP5, the nearest genes were *GRMZM2G057159* and *GRMZM2G381691*, which located downstream 5120 bp to SNP4 and upstream 52369 bp to SNP5 (Table 2). In these two locations, the LD decay were both larger than 0.8.

**Table 2:** Annotation of the candidate genes related to the five SNPs associated with husk traits.

Candidate genes	Trait^a^	Chr	Gene interval(bp)	Distance from the related SNP to the edge of the gene (bp)^b^	Annotation
GRMZM2G003984	HN	1	48953687–48999879	+30602	Lon protease
GRMZM2G012416	HN	4	204054112–204064316	−29974	ARM repeat superfamily protein
AC212835.3_FG007	HT	2	21007540–21010895	Located	Poly(U)-specific endoribonuclease-B
GRMZM2G057159	HT	2	221366850–221371447	+5120	Subtilase family protein
GRMZM2G381691	HT	10	94248710–94251264	−52369	CCT domain protein

^a^Husk layer Number (HN) and Husk Thickness (HT).

^b^The positive (+) and negative (−) values represent related SNPs location in the 5′ and 3′ direction, respectively, of their candidate gene.

## Variance of SNPs in five candidate genes

**Figure 4:**
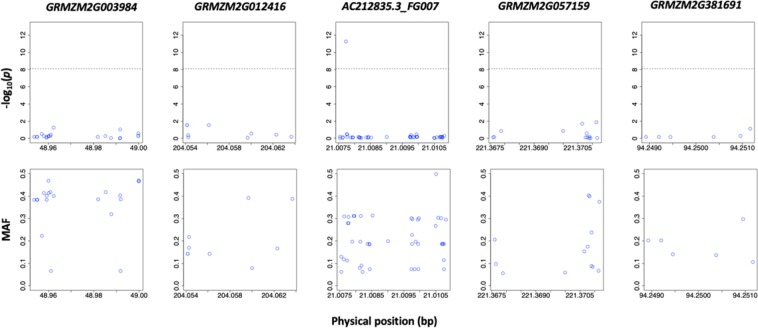
Variances of SNPs for *P*-value associated with husk traits and MAF in five candidate genes. On the half above, the *P*-values were calculated using BLINK software. The horizontal black dashed line represents the Bonferroni multiple test threshold corresponding to a type I error of 1% (*p* < 7.98 × 10^−9^). On the half blow, the *y* axis represents the Minor Allele Frequency (MAF). The *x* axis represents the physical position of each candidate gene.

To detect the variance of SNPs in five candidate genes, we displayed the MAF and P-value of all SNPs for each candidate gene (Fig. 4). The number of SNPs were 22, 9, 45, 13 and 6 in five candidate genes. For MAF, the widest distribution appeared in *AC212835.3_FG007* from 0.062 to 0.0498. The narrowest distribution appeared in *GRMZM2G381691* from 0.106 to 0.297. For *P*-value associated with husk traits, the widest distribution appeared in *AC212835.3_FG007* from 0.018 to 11.270. The significant SNP located in this gene caused the pick. In other four candidate genes, the SNPs showed narrower distribution than the former. BLINK is different with MLM. If one significant SNP pass the threshold, other SNPs in same LD won’t pop up. So, if the significant SNP didn’t locate in the candidate gene, no point above the threshold could be found.

### Candidate gene expression profile

**Figure 5:**
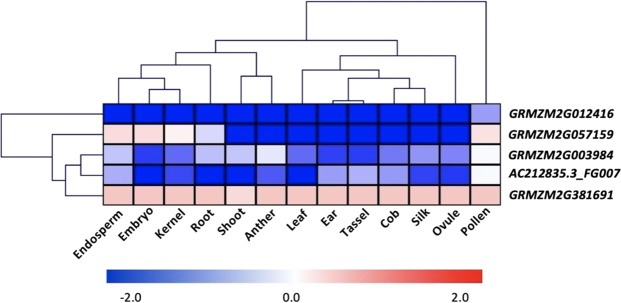
Heat-map of tissue-specific expression patterns of the five candidate genes. The in-silico gene expression of the six candidate genes were extracted for husk and other 13 tissues. The gene expressions were represented by the normalized Reads Per Kilobase per Million (RPKM). The log2 transformation of the ratio of gene expression in husk against the gene expression in other 13 tissues were hierarchically clustered in two dimensions (gene and tissue) and displayed using heat map. The expression of *GRMZM2G012416* in husk were lower than all other tissues.

To determine whether these genes exhibit tissue-specific expression patterns, we performed an in-silico expression pattern analysis using published RNA-seq datasets from 14 different organs/tissues, including husk^8,27–30^. The dataset used in this analysis is listed in Table S1. Gene *GRMZM2G012416* associated with HN, showed lower expression in husk tissue relative to all other tissues. Gene *GRMZM2G057159* associated with HN, genes *GRMZM2G003984* and *AC212835.3_FG007* associated with HT, showed lower expression in husk tissue relative to partial other tissues. Gene *GRMZM2G381691* associated with HT, showed little higher expression in husk tissue relative to all the other tissues. In addition, according to these husk candidate gene expression patterns, the 13 tissues can be categorized into 2 groups. The pollen tissues clustered into the first group and other tissues clustered into the second group (Fig. 5).

We also conducted the distribution of correlation between gene expression and husk traits. Gene *GRMZM2G012416* and *AC212835.3_FG007* the highest negative and positive correlation for HN and HT compared with all the other genes. But no candidate gene pass the significant level (*p* < 0.05) than other genes. The probable reason was RNA-seq had tissue-special feature. This RNA-seq data derived by kernel and not husk.

## Discussion

Husk traits are controlled by multiple genes or QTL, plus they exhibit wide phenotypic variation with normal distributions within the studied association populations^20–22^. Herein, GWAS was chosen as a suitable method to detect the genomic basis of husk traits. However, previous GWAS studies found no significant SNPs using the stricter Bonferroni-corrected threshold of α = 0.01 (*P* < 7.98 × 10^−9^)^21,22^, indicating the complexity of molecular regulation and the limitations of the previous analysis methods for maize husk morphology.

### Enhancing marker density benefited GWAS detection of significant SNPs for husk traits

The genome is larger and the LD decay rate is faster in maize compared to a number of other plant species^31,32^. Thus, the minimum number of markers required for successful GWAS in maize is 0.5–1.0 million^33^. In the 508-line association panel, Yang *et al*. (2014) combined data from two genotyping platforms (RNA-seq and 50 K SNP array)^26^. Our previous study^21^ used these ~ 0.55 million markers to identify 9 significant SNPs by MLM with a corrected cutoff of *P* < 1.04 × 10^−5 ^^34^. Liu *et al*. increased the marker density of this association panel to ~1.25 million markers by combining GBS and 600 K SNP array into a whole genetic map^25^.

Using these combined ~1.25 million markers by MLM, we didn’t find significant SNPs associated with husk traits, using the most stringent Bonferroni-corrected threshold of α = 0.01. Compared to sparse markers by BLINK, we found five significant SNPs associated with two husk traits, using the former stringent threshold. That is, not one of these new SNPs overlapped the significant SNPs identified for HL, HN, HT, or HW in either of our previous GWAS^21^ or linkage mapping study^20^. One major reason is that most of these SNPs were detected from new genotype platforms, which were unavailable when the previous studies were performed.

Admittedly, Bonferroni-corrected thresholds have been criticized as overly conservative and have reduced statistical power for finding significant SNPs^35^. Specifically, these thresholds may preclude the identification of existing loci with smaller effects^36^. Nevertheless, the Bonferroni-corrected threshold remains an efficient standard for controlling Type I errors (detecting false positives when the null is true) and avoiding spurious conclusions in GWAS^23^.

### BLINK improved GWAS of husk traits

False positives can also be controlled by improving statistical methods. A MLM that incorporates population structure and kinship will control inflation well in GWAS^37^. However, for some complex traits associated with population structure, such as *Arabidopsis* flowering time, this method may also remove signals of known genes as background noise^38^. To solve this problem in GWAS, the new statistical method, BLINK, uses a multiple loci test method instead of a single loci test method for MLM, by combining a fixed effect model (FEM), Bayesian information criteria, and linkage disequilibrium information^24^. Compared to MLM, BLINK improves statistical power (defined as the proportion of QTN detected for a specific level of TYPE I error) in both real and simulated data^24^.

So far, only two GWAS results have been published for maize husk traits^21,22^. In this current study, our SNP marker density was two times more than Cui *et al*.^21^ and 400 times more than Zhou *et al*.^22^ (Table S2). By using BLINK instead of MLM in our GWAS, computing time per trait was only 50 seconds, which is 216 times faster than Cui *et al*.^21^ and 2 times faster than Zhou *et al*.^22^. Particularly, even with the most severe Bonferroni-corrected threshold of α = 0.01, BLINK with dense and sparse markers found five and one significant SNPs associated with husk traits, whereas no significant SNPs were found in the other two studies using the same threshold.

### Novel candidate genes associated with husk traits

In our previous GWAS study with the 508-line association panel, both MLM and GLM models detected 63 candidate genes associated with husk traits^21^. In our most recent study that combined association analysis and linkage mapping, we found four candidate genes for HL and one candidate gene for HN^20^. These candidate genes were clustered into multiple functional categories, including cellular trafficking, transcriptional regulation, signal transduction, and metabolism.

In this study, according to LD decay of each significant SNP, we identified five novel candidate genes corresponding to the five associated SNPs (Table 2). For HN, we identified two candidate genes.*GRMZM2G003984* encoded a Lon protease, which can degrade misfolded proteins or some specific regulatory proteins involving in mitochondrial biogenesis during seedling establishment^39^ and cell death in plant^40^. *GRMZM2G012416* encodes an Armadillo (ARM) repeat protein. ARM-repeat proteins are motifs that mediate protein-protein interactions involving various animal proteins^41^. In Arabidopsis, a large amount of ARM-repeat proteins is reported as members of the U-Box E3 ubiquitin ligase family and involved in GA signaling or regulating mRNA levels in pathogen responses. These candidate genes implied that speed of cell death and GA signal may play important role in husk layer number.

For HT, we identified three candidate genes. *AC212835.3_FG007* encodes a Poly(U)-specific endoribonuclease-B. Poly(U)-specific endoribonuclease was first found from calf thymus but no reports in plant^42^. *GRMZM2G057159* encodes a subtilase family protein, which also named subtilisin-like serine proteases family protein. Two subtilase genes, HvSBT3 and HvSBT6, were postulate as key components of senescence-associated proteolysis in barley^43^. *GRMZM2G381691* encodes a CCT domain protein. *ZmCCT* played important role of affecting photoperiod response in maize^44^. The higher expression of *ZmCCT* alleles from tropical maize under long day lengths will show later flowering than temperate maize alleles. Congruously, our previous study reported that the HT in tropical subgroup is significantly thicker than other temperate subgroup^21^.

## Materials and Methods

### Plant materials and phenotyping

The association panel was comprised of 508 maize inbred lines that were globally collected from tropical, subtropical, and temperate germplasms^45,46^. According to population structure, all 508 inbred lines were clustered into four subgroups: stiff stalk (SS), non-stiff stalk (NSS), tropical-subtropical (TST), and mixed (MIX). About 10 lines were treated as missing data due to poor germination at each planting location. Four husk traits, HL, HN, HT, and HW, were measured at the same stage of maturity, at the same time, in two locations in China: Hainan (HN) in 2014 and Beijing (BJ) 2015. Detailed husk measurement information has been described in Cui *et al*.^21^.

In our previous husk GWAS study^21^, Best Linear Unbiased Predictions (BLUPs) were used as the response variable. The phenotypic distribution of all husk phenotypes was similar to our previous report (Fig. S4). In this study, we found that correlations *(r*^2^) between means and BLUPs were > 0.95 for each husk trait. This is consistent to previous finding^47^ that mean values and BLUPs are similar with balanced data when individual lines were treated as unrelated. Therefore, we used the mean values instead of BLUPs.

### Genotyping, integrated mapping, and imputation

To obtain higher marker density, four genotyping platforms were used, the Illumina Maize SNP50 array, RNA sequencing, GBS, and the Affymetrix Axiom Maize 600 K array. RNA sequencing was performed on developing kernels at 15 days after pollination for 368 out of the 508 maize inbreds^48^. To add missing genotypes into the additional 140 inbreds, which were only genotyped by a SNP-chip, Yang *et al*. expanded this association panel size using a two-step data-imputation method^26^. This method combines the identity by descent (IBD) based projection and k-nearest neighbor (KNN) algorithm.

Ultimately, they obtained 0.55 million SNPs for all 508 lines. In two recent studies, 469 lines used GBS^49^ and 153 lines used the 600 K SNP array for further genotyping^50^. In total, 670,411 and 502,824 SNPs were found by the GBS and 600 K genotyping platforms, respectively. After strict quality control procedures for each dataset, the genotypes from four different genotyping platforms were merged. Beagle v4.0^51^ was then used to perform genotype imputation. Finally, the integrated map obtained 1.25 M SNPs with MAF ≥ 5%. The final, merged genotyping set can be downloaded from www.maizego.org/Resources. The 1.25 M SNPs were selected as dense markers. Then we intersected the 0.55 M and 1.25 M SNPs to obtain 0.47 M SNPs as sparse markers. Thus, all the sparse markers were included in dense markers.

### Association analysis

The 1,253,814 SNPs and 474,972 SNPs (MAF ≥ 0.05) were selected as dense and sparse markers for GWAS by combining the data from four genotyping platforms (RNA-seq, 50 K SNP array, 600 K SNP array, and GBS) and two genotyping platforms (RNA-seq, 50 K SNP array)^25,48^. Association analysis for four husk traits was conducted with Bayesian information criterion and Linkage-disequilibrium Iteratively Nested Keyway (BLINK)^24^. The BLINK package can be downloaded from https://github.com/Menggg/BLINK. The first three PCs were treated as covariates to perform GWAS. We used the standard Bonferroni-corrected threshold of α = 0.01 as the significance cutoff. The suggested *P*-value was computed as 0.01/n (n = 1,253,814 or 474,972), and we obtained the *P*-value of 7.98 × 10^−9^ and 2.11 × 10^−8^ as the final significance cutoff in the association analysis. GWAS by MLM with dense markers was performed in GAPIT 2.0 software^52^. The first three PCs and threshold were as same as previous data using by BLINK. The kinship was calculated with dense and sparse markers by GAPIT 2.0.

The contribution of identified SNPs to the phenotypic variance was estimated using anova() function in the R package. Taking the first three PCs into account, ﻿the R^2^ of each significant SNP, were calculated by the linear models:

1 $$Y=\alpha X+\beta \cdot P+\varepsilon $$

The total variance of all significant SNPs was calculated by the linear models:

2 $${\rm{Y}}=\alpha \mathop{\sum }\limits_{i=1}^{m}Xi+\beta P+\varepsilon $$

where *Y* and *X* represent the phenotype and SNP genotype vectors, respectively; *P* is the matrix of the first three PCs; *α* is the SNP effect; *β* is the subpopulation effects; *ε* is the random effects.

## **LD decay surrounding the candidate QTNs**

LD was calculated for each candidate QTN within its surrounding regions (1 Mb).

LD value equals the Pearson correlation of the genotype for one surrounding SNP and that for the candidate QTN.

## **Distribution of correlation between candidate genes and husk traits**

RPKM of RNA-seq data was download from www.maizego.com. After the 15^th^ days of pollination, the kernel of 368 association panel (a part of 508 association panel) was sequenced by RNA-seq. Based on RPKM, RNA-seq reads was computed and scaled. ﻿After RPKM normalization by edgeR package, including all genes with a median expression level more than zero, the whole distribution of expression level for every gene in this panel was normalized by a normal quantile transformation. We calculated the pearson correlations between each gene’s expression level and the husk traits of 368 individuals. For the gene expression level, the RPKM values were used.

## **Heat-map of candidate genes**

All RNA-Seq datasets from 14 maize tissues (including anther, cob, ear, embryo, endosperm, husk, kernel, leaf, ovule, pollen, root, silk, shoot, and tassel) were downloaded from NCBI’s Sequence Read Archive (SRA) database. The SRA sample ID and related reference for all tissues are listed in Table S1. Performing the TopHat pipeline, RNA-Seq reads were mapped to the *AGPv2* with the built-in Bowtie mapping program. Only the unique mapped reads were retained for counting Normalized Reads Per Kilobase Million **(**RPKM) by Cufflinks software. To categorize the pattern of gene expression amount tissues, we derived the log2 transformation on ratio of normalized RPKM in husk against the normalized RPKM in other tissues. Values greater than +2 or less than −2 were adjusted to 2 or −2, respectively.

## Supplementary information

Supplementary information
