# Supplementary material for: Denser Markers and Advanced Statistical Method Identified More Genetic Loci Associated with Husk Traits in Maize
Source: Sci Rep. 2020 May 18;10:8165. doi: 10.1038/s41598-020-65164-0 (PMC7235265; doi:10.1038/s41598-020-65164-0)
Supplement: Supplementary file 1 — Supplementary information [file 41598_2020_65164_MOESM1_ESM.docx]

**Supplementary**

**Denser Markers and Advanced Statistical Method Identified More Genetic Loci Associated with Husk Traits in Maize**

Zhenhai Cui^1,2#^, Haixiao Dong^2,3#^, Ao Zhang^1^, Yanye Ruan^1^, Siqi Jiang^1^,

Yan He^4*^ and Zhiwu Zhang^2*^

^1^College of Biological Science and Technology, Liaoning Province Research Center of Plant Genetic Engineering Technology, Shenyang Key Laboratory of Maize Genomic Selection Breeding, Shenyang Agricultural University, Shenyang 110866, China

^2^Dept. of Crop and Soil Sciences, Washington State University, Pullman, WA 99164, USA;

^3^College of Plant Sciences, Jilin University, Changchun 130062, China

^4^National Maize Improvement Center of China, Beijing Key Laboratory of Crop Genetic Improvement, China Agricultural University, Beijing, 100094, China

**Table S1. The ratio of Normalized Reads Per Kilobase Million (RPKM) for five candidate genes dividing in husk^a^ by in other tissues ^b-e^**

| Sample ID | Gene | *GRMZM2G003984* | *GRMZM2G012416* | *AC212835.3_FG007* | *GRMZM2G057159* | *GRMZM2G381691* |
| --- | --- | --- | --- | --- | --- | --- |
| SRR1169636 | Endosperm^b^ | -1.613 | -6.437 | -2.041 | 0.292 | 0.457 |
| SRR1168430 | Embryo^b^ | -0.478 | -6.100 | -0.664 | 0.292 | 0.457 |
| SRR1170941 | Kernel^b^ | -1.212 | -5.963 | -1.509 | 0.109 | 0.457 |
| SRX012381 | Root^c^ | -0.456 | -4.571 | -2.494 | -2.201 | 0.299 |
| SRX012380 | Shoot^c^ | -0.509 | -4.498 | -2.399 | -0.299 | 0.457 |
| SRR189769 | Anther^d^ | -1.214 | -6.384 | -2.583 | -2.152 | 0.457 |
| SRR504466 | Leaf^e^ | -1.577 | -6.729 | -0.778 | -2.502 | 0.457 |
| SRR504476 | Ear^e^ | -1.619 | -6.754 | -0.624 | -2.405 | 0.457 |
| SRR504471 | Tassel^e^ | -1.088 | -5.969 | -0.823 | -3.433 | 0.457 |
| SRR189763 | Cob^d^ | -0.848 | -6.399 | -1.571 | -3.555 | 0.457 |
| SRR189772 | Silk^d^ | -0.181 | -3.505 | -1.366 | -2.526 | 0.457 |
| SRR189770 | Ovule^d^ | -0.983 | -5.835 | -1.658 | -5.158 | 0.457 |
| SRR189771 | Pollen^d^ | -0.055 | -0.792 | -0.051 | 0.232 | 0.457 |

a Data is reported by Wang et al., 2013

b Data is reported by Chen et al., 2014

c Data is reported by Wang et al., 2009

d Data is reported by Davidson et al., 2011

e Data is reported by Bolduc et al., 2012

References

Jian Chen, Biao Zeng, Mei Zhang, Shaojun Xie, Gaokui Wang, Andrew Hauck, Jinsheng Lai. Dynamic Transcriptome Landscape of Maize Embryo and Endosperm Development. Plant Physiol. 2014, 166 (1) 252-264

Bolduc N, Yilmaz A, Mejia-Guerra MK, Morohashi K, O'Connor D, Grotewold E,
Hake S. Unraveling the KNOTTED1 regulatory network in maize meristems. Genes Dev. 2012, 1;26(15):1685-90

Davidson RM, Hansey CN, Gowda M, Childs KL, Lin H, Vaillancourt B, Sekhon RS, de Leon N, Kaeppler SM, Jiang N, Buell CR. Utility of RNA Sequencing for Analysis of Maize Reproductive Transcriptomes. Plant Genome J., 2011, 4:191-203

Wang X, Elling A A, Li X, et al. Genome-Wide and Organ-Specific Landscapes of Epigenetic Modifications and Their Relationships to mRNA and Small RNA Transcriptomes in Maize. Plant Cell, 2009, 21(4):1053-1069

Wang P, Kelly S, Fouracre JP, Langdale JA. Genome-wide transcript analysis of early maize leaf development reveals gene cohorts associated with the differentiation of C4 Kranz anatomy. The Plant Journal. 2013, 75(4):656-670.

**Table S2. Comparison among different GWAS of husk traits.** At present, only two previous GWAS related to husk traits have been published, Cui et al. 2016 and Zhou et al.2016.

|  | This study | Previous Cui et al. | Previous Zhou et al. |
| --- | --- | --- | --- |
| Husk traits^a^ | HL, HN, HT, HW | HL, HN, HT, HW | HN, husk weight |
| Markers | 1.25 Mb | 0.55 Mb | 3.07 Kb |
| Individuals | 508 | 508 | 253 |
| GWAS methods^b^ | BLINK | TASSEL (MLM) | TASSEL (MLM) |
| Computing time per trait | ~30 seconds | ~3 hours | ~1 minutes |
| Significant SNPs (0.01/n)^c^ | 6 | 0 | 0 |
| *P* value of the most significant SNP in HN | 1.58E-14 | 2.57E-06 | ﻿2.29E-06 |

^a^ Husk Length (HL), Husk layer Number (HN), Husk Thickness (HT), Husk Width

^b^ BLINK, Bayesian-information, and Linkage-disequilibrium Iteratively Nested Keyway (Huang et al. 2018); TASSEL, Trait Analysis by aSSociation, Evolution, and Linkage; MLM, the mixed linear model. MLM is one of the analysis models in TASSEL (Bradbury et al. 2007; Yu et al. 2006).

^c^ 0.01/n, the Bonferroni multiple test threshold corresponding to type I error of 1%; n represents the number of makers.


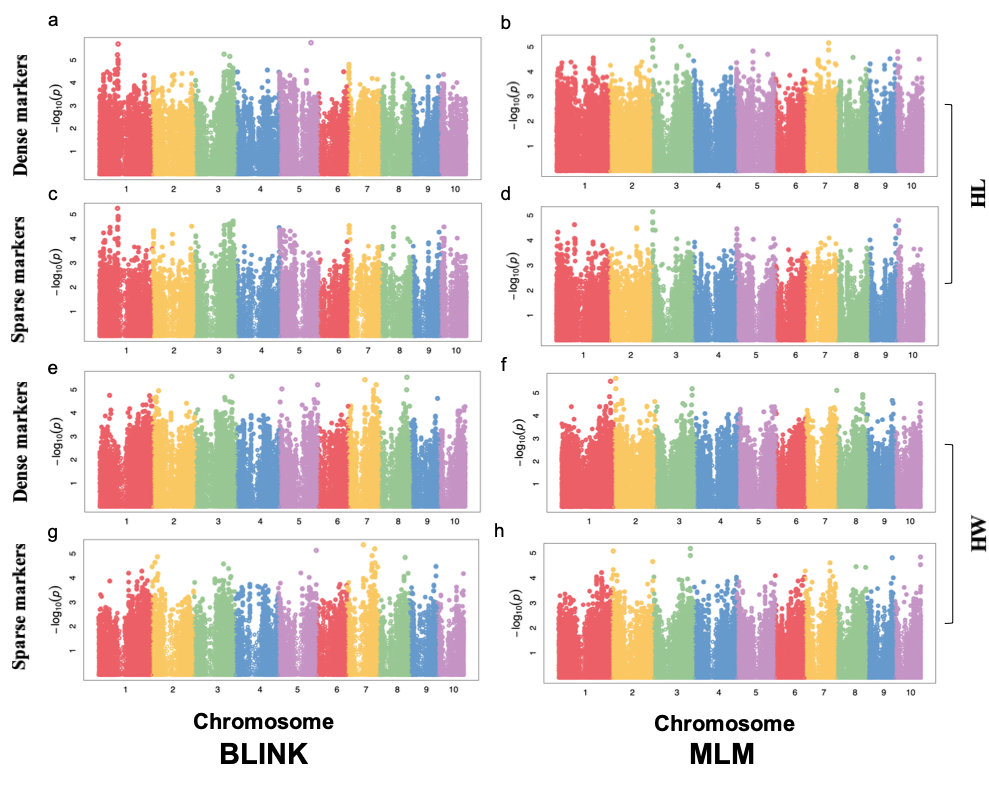


**Figure S1. Manhattan plots with dense and sparse markers by BLINK and MLM of husk layer length and husk width in maize**. The Manhattan plots on the left panel display the *P-*values of the SNPs, categorized by chromosome and position along the chromosome, associated with Husk Length (HL) and Husk Width (HW). The *P*-values were calculated using BLINK and MLM software. No SNPs (purple circles) were identified as significantly associated with HT and HW. (**a)** Mahattan plot of HL with dense markers by BLINK. (**b**) Mahattan plot of HL with dense markers by MLM. (**c**) Mahattan plot of HL with sparse markers by BLINK. (**d**) Mahattan plot of HL with sparse markers by MLM. (**e**) Mahattan plot of HW with dense markers by BLINK. (f) Mahattan plot of HW with dense markers by MLM. (g) Mahattan plot of HW with sparse markers by BLINK. (h) Mahattan plot of HW with sparse markers by MLM.


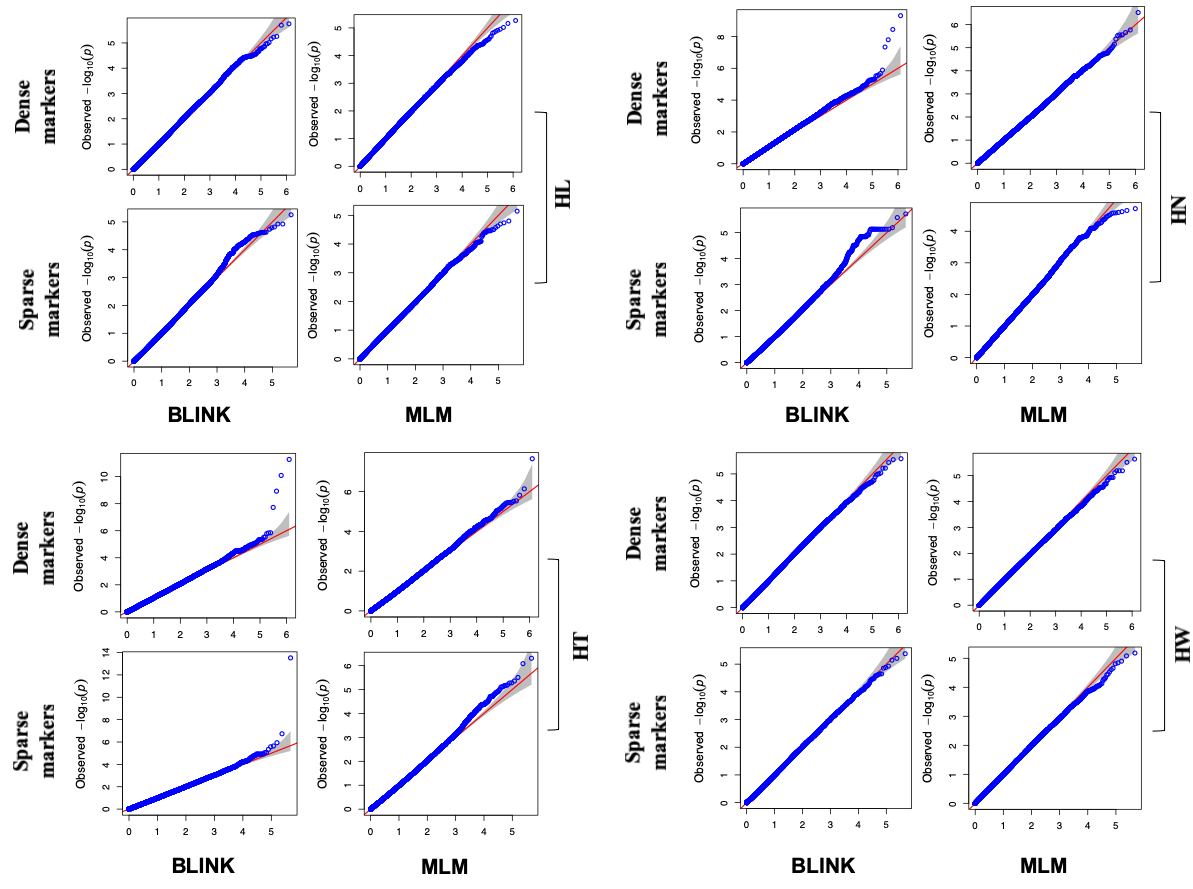


c

d

b

a

Figure S2. Quantile-quantile (QQ) plots with dense and sparse markers by BLINK and MLM of four husk traits. The QQ plots exhibit the observed probabilities (blue) that exceeded the expected probabilities (red). (a) Husk Length (HL). (b) Husk layer Number (HN). (c) Husk thickness (HT). (d) Husk Width (HW).


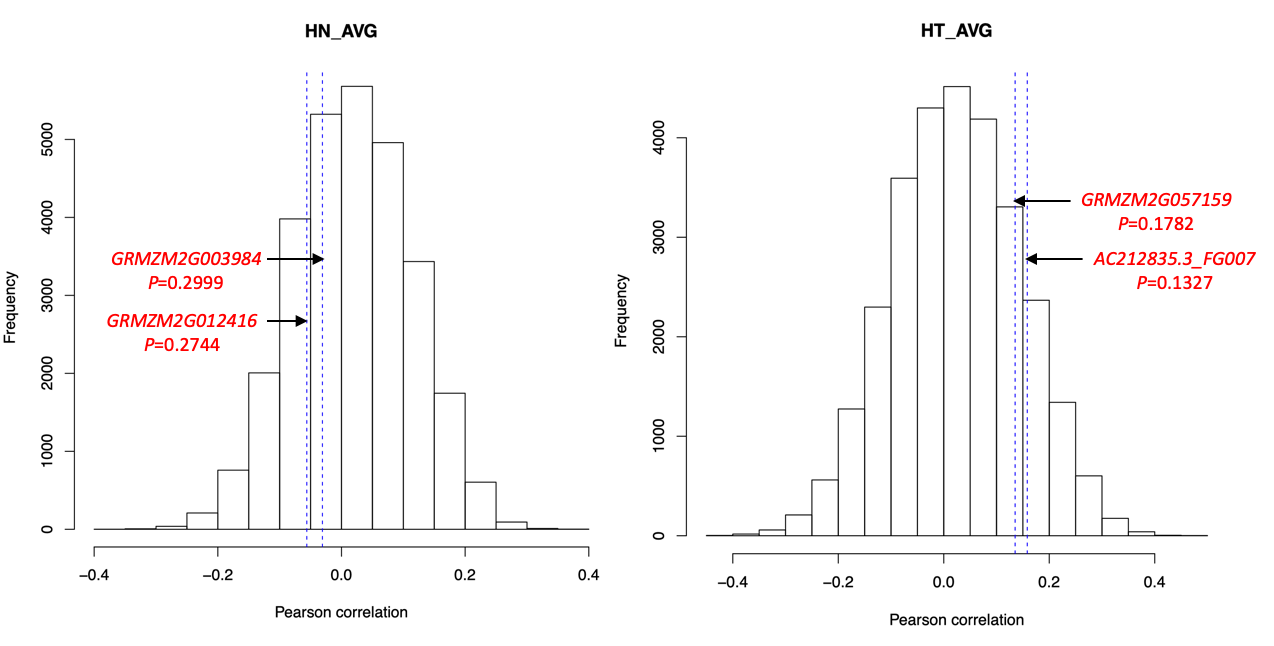


a

b

**Figure S3. Distribution of correlation between the gene expression of all genes and phenotype of husk. (a)** Husk layer number (HN). (**b**) Husk thickness (HT). The blue dash line represents the correlation between significant candidate genes and phenotype. The red words represent the four candidate genes and its *P*-value associated with the gene expression of all the other genes. One candidate gene of HT, *GRMZM2G381691*, doesn’t include in this RNA-seq data.


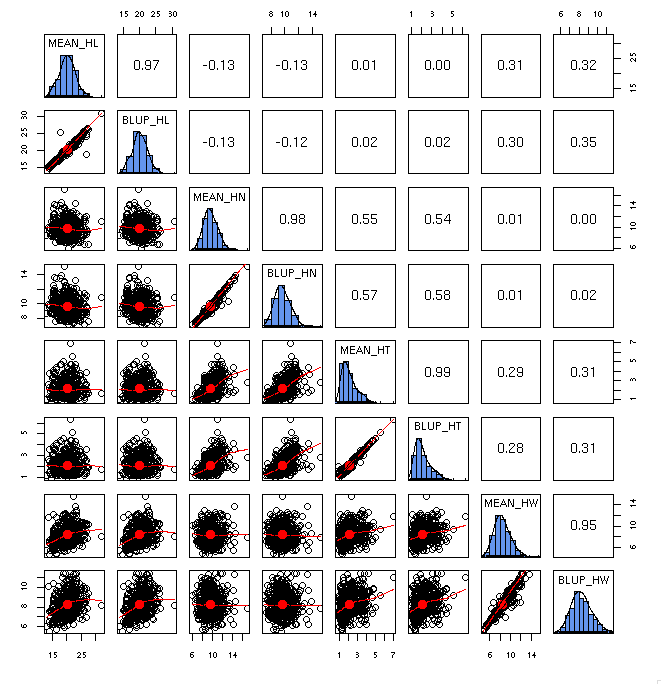


**Figure S4.** **Correlations and frequency distributions of four husk traits between mean and Best Linear Unbiased Prediction (BLUP).** The four husk traits were husk length (HL), husk layer number (HN), husk thickness (HT) and husk width (HW). The mean was calculated across replicates and locations. BLUPs were obtained from our previous study (Cui et al. 2016). The plots on the diagonal line exhibit the phenotypic distribution of the mean and BLUP for each trait. Displayed below the diagonal line, are the scatter plots for mean and BLUP values; displayed above the diagonal line are Pearson correlation coefficients. The red line and red dot represent the lowest regression fitting curve and the correlation ellipse, respectively.
